# Introducing New Paths towards Public Primary Healthcare Services in Greece: Efforts for Scaling-Up Mental Healthcare Services Addressed to Older Adults

**DOI:** 10.3390/healthcare10071230

**Published:** 2022-06-30

**Authors:** Ourania Pinaka, Fotios Gioulekas, Evlampia Routa, Aikaterini Delliou, Evangelos Stamatiadis, Ioanna Dratsiou, Evangelia Romanopoulou, Charalambos Billinis

**Affiliations:** 1 4th Local Primary Healthcare Unit of Ampelokipi Larissa, 5th Regional Health Authority of Thessaly & Sterea, 5–7 Myron, 41447 Larissa, Greece; evirouta@hotmail.com (E.R.); katedell9@gmail.com (A.D.); 2 Faculty of Public and One Health, School of Health Sciences, University of Thessaly, 43100 Karditsa, Greece; billinis@uth.gr; 3 5th Regional Health Authority of Thessaly & Sterea, Mezourlo Area, 41110 Larissa, Greece; fogi@dypethessaly.gr (F.G.); vstam@dypethessaly.gr (E.S.); 4 Medical Physics Laboratory, School of Medicine, Aristotle University of Thessaloniki, 54124 Thessaloniki, Greece; idratsiou@gmail.com (I.D.); evangeliaromanopoulou@gmail.com (E.R.)

**Keywords:** primary health care, primary care providers, mental health disorders, training, psychometric and cognitive assessment

## Abstract

The exponential growth in the aging population challenges the Primary Care Providers (PCPs) who provide health care services to older adults who are considered highly vulnerable and are in need of specialized healthcare services. The development of new policies and the adoption of appropriate health strategies by PCPs may improve the early detection and prevention of mental disorders in older adults. This reduces both queuing and costs in outpatient clinics while preventing stigma for patients and families. To this end, specialized training for PCPs at the Local Primary Health Care Unit (LPHCU) was provided in order to conduct efficient assessments of older adults (65 and above years old, without previously diagnosed depression or dementia, and willing to participate). The assessment is based on the Mini Mental State Examination (MMSE) and Geriatric Depression Scale (GDS). Older adults identified with MMSE <20 and GDS >5 were referred to the psychiatric outpatient clinic. The aim of this study is to discuss evidence-informed policymaking in Greece with a focus on advancing mental health practices and scaling up quality primary healthcare services for older adults.

## 1. Introduction

People live longer and healthier lives as a result of advancements and improvements in healthcare over the last century [1]. However, the rise in life expectancy and the aging of the global population have been associated with an alarming increase in the worldwide burden of chronic and non-communicable diseases (NCDs) [2], including obesity, diabetes, heart disease, stroke, cancer, and mental health disorders [3], imposing a major impact on healthcare and social policy planning [4]. Mental health is considered an integral part of health and well-being. People living with mental health disorders and psychosocial disabilities often experience significantly greater rates of disability and mortality [5]. In fact, based on the increasing prevalence of mental disorders in older adults, with the most frequently diagnosed among them being depression and anxiety [6,7], maintenance of the quality of life, functional capacity, and social participation of older adults is set on the center of attention [8]. Indeed, in the last decade, mental health has received increased attention worldwide from governments, nongovernmental organizations (NGOs), and multilateral organizations resulting in setting the groundwork for the provision of quality care and support through the establishment of person-centered, human rights-based, and recovery-oriented care and services [9].

Integrating specialized health services—such as mental health services—into Primary Health Care (PHC) has been, for a long time now, one of the World Health Organization’s (WHO) most fundamental health care recommendations in order to increase identification in the first stage of the mental disorders [10]. Now, in the context of improving access to care and service quality [5], the WHO recommends the development of comprehensive community-based mental health and social care services [9]. The benefits of the integration, both for patients and for the health system, are many and important: (a) improved access to health, (b) improved prevention and detection of mental disorders, (c) reduced costs and waiting times, (d) conserved human and material resources, (e) reduced stigma for people and their families, (f) increased self-confidence for providers, and (g) continuity of care [11]. Greece exemplifies one of the numerous countries that have been adversely affected as a result of the financial crisis and austerity measures leading to a poor Primary PHC and a lack of substantial synergies in the context of integrated care provision [12]. The aim of this study is to discuss evidence-informed policymaking in Greece with a focus on advancing mental health practices and scaling up quality primary healthcare services for older adults.

## 2. Current Status in Greece

According to recent demographic data in 2015, the proportion of the 65+ and 85+ years people was 20.9% and 2.8%, respectively. It is projected to accordingly reach 27.9% and 4.5% in 2035 [13]. As already highlighted, mental health problems may highly impact older adults’ ability to carry out basic daily living activities, reducing their independence and quality of life and, therefore, access to fundamental human rights [14]. The first step to reducing these negative consequences is through diagnosis. Unfortunately, mental health problems remain undiagnosed and untreated, due to the difficulty in the identification of the symptoms. Moreover, the adequate provision of help and support to older adults deteriorates the problem [15].

Notable efforts on reforming the Greek PHC by introducing policies and practices on the National Primary Health Care Network and the Greek National Health Service have been implemented. Indeed, PHC is considered the appropriate field for the prevention and early identification of mental disorders in older adults. Particularly, the introduction of LPHCUs was at the core of the PHC reform aiming to provide holistic and high-quality patient-centered care [16]. However, the Greek PHC system has been deemed ineffective, with flaws in both the structure of primary care with regard to economic conditions and capacity building, as well as in the service delivery mechanisms in terms of access, continuity, and comprehensiveness of primary care [17]. The application of assessments for the early detection of mental disorders in the population of older adults is not performed on a regular basis. Time constraints, lack of staff in PHC, lack of educated and trained providers, and the reluctance of professionals to take such initiatives are the main factors for not using the associated identification tools or methods.

Despite the above challenges, a deeper understanding of the fundamental concept of providing integrated specialized healthcare services is more challenging than ever before. Therefore, the synergy between the PHC sector and specialized professionals in mental health care will adequately serve the needs of older adults. The training of the PCPs in the implementation of neuropsychological assessments by specialized professionals proved to be essential. It led to the early recognition of the disorders in older adults [18] and defined a clear and fast pathway from primary care (PHC professionals) to tertiary care (mental health specialists).

## 3. Proposing a Reformation of the Existing Public Primary Health Care

### 3.1. Establishing Specialized Structures for Health Care Services

In 2017, the Greek LPHCUs were established, which are considered to be important components of the newly designed primary health care system and serve as the area’s initial point of contact for residents. In these units, multidisciplinary teams (general practitioners, nurses, health visitors, and social workers) provide PHC services for people in the community. It is denoted that there was no provision for healthcare professionals working in the field of mental health services. Therefore, it is recommended that through specific training and education programs the existing personnel could be scaled-up and be linked with specialized mental-health professionals.

### 3.2. Organizing Training Sessions

The high percentage of older adults attending an LPHCU, the increase of mental disorders, and the difficulty in recognizing mental disorders, due to older adults’ clinical picture and the existence of other comorbidities, were communicated to the Psychiatric Clinic of the University Hospital of Larissa. Consequently, it was decided to schedule relevant training for the PCPs. Therefore, four training sessions were organized for healthcare professionals in LPHCUs and took place in November 2018. It should be noted that due to the COVID-19 crisis, the scheduled training follow-up was postponed. The two-fold aim of the training sessions was to improve PCPs’ skills and knowledge in the field of mental health and to increase the interconnection between the LPHCUs. Supervised by a healthcare professional, the two-hour training sessions comprised of lectures on dementia, depression symptoms/treatment, as well as hands-on training for the use of screening tests for evaluating the cognitive condition of the older adults.

### 3.3. Focusing on Implementing a New Framework

The PCPs who participated in the training sessions were responsible for conducting the psychometric and cognitive assessments of the older adults. Thus, the training sessions acted as an implementation paradigm of mental health services in primary care and, more specifically, in the LPHCU. Older adults who were already registered in the LPHCUs and were being monitored for other health problems by the unit’s General Practitioners (GP) were eligible to receive mental health services. Older adults who did not have a prior recorded history of dementia or depression and came to the unit upon a scheduled appointment could be managed either by those interested or by their relatives.

**Figure 1:**
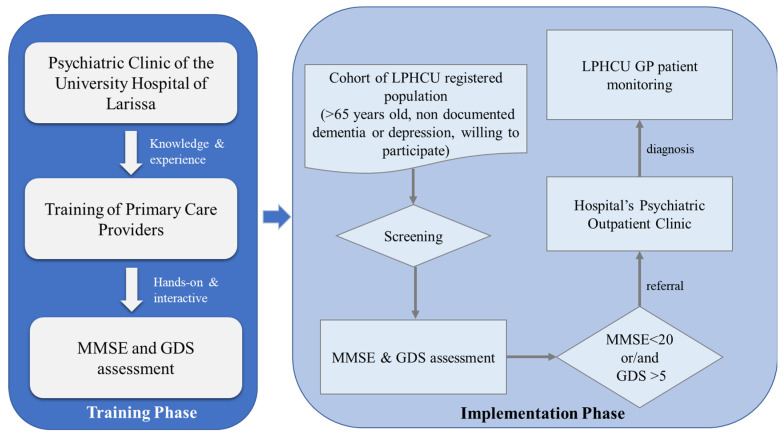
Integration path between the hospital’s Psychiatric clinic and workflow to identify and monitor patients with mental disorders.

Figure 1 shows the training phase along with the implementation phase of the protocol used for the identification of patients with mental disorders, the referral to the psychiatric outpatient clinic, and the monitoring. During the tanning period, the experience and knowledge of the Psychiatric clinic of the University Hospital of Larissa, regarding the identification of symptoms and signs of mental disorders, were transferred to the PCPs through several sessions. In this interaction, PCPs were trained on how to efficiently conduct MMSE, the GDS assessments, and triage patients.

Therefore, the MMSE [19] and the GDS [20] assessments were used to examine participants’ mental health and wellbeing by the unit’s qualified healthcare professional during the implementation phase. These assessments have been validated in clinical practice [19,20] and comprise an identification tool for mental disorders, well-deployed in psychiatric outpatient clinics. The assessments were translated into Greek, as provided by the specialists. Furthermore, a short medical history was collected by the GP of the unit for each of those patients, which included chronic illnesses, medications taken, and the results of their assessments. Older adults over 65 years old, with an MMSE score of lower than twenty and a GDS score of greater than five, were referred to the Psychiatric outpatient clinic of the University Hospital of Larissa along with the aforementioned data collected by the GP. After the diagnosis, an informative note from the psychiatrist was sent to the GP in order to provide a follow-up about the patient. These patients continued to be monitored by the GP.

## 4. Implication of Mental Health Care in Primary Health Care

The unprecedented increase in the aging population in recent decades has led to an increased prevalence of chronic diseases and mental health disorders posing major considerations for the health system globally [18]. Treating mental health disorders in older adults by PCPs is a challenge due to the fact they (a) develop different symptoms, such as tiredness or weight loss, and are often interpreted as an expected process of aging in older adults [21]; (b) comprise a field of knowledge in which the majority of PCPs are not properly trained in identifying or providing care to older adults with mental disorders [14]; (c) lead to stigma in terms of mental health [22]; and (d) restrict the independence of older adults [23,24].

Despite the reported needs, preventive interventions for the support of the older adults’ mental health have not been adequately developed in the context of psychiatrics in Greece, and significant deficiencies in the provision of care for older adults have been recorded [21]. In this study, evidence-informed policymaking in LPHCU in Greece is proposed with a focus on advancing mental health practices and scaling-up quality primary healthcare services for older adults. The implementation of mental health services, as described above, through appropriate psychometric and cognitive assessments in PHC could potentially improve (a) the interconnection and collaboration between a primary and a tertiary structure, (b) the training of PCPs, (c) the application of a well-established referral system, and (d) the operation of an adequate recording system with a feedback mechanism that efficiently assists with the early identification of people that previously were non-diagnosed. Furthermore, this approach could relieve secondary/tertiary care facilities from a variety of cases, thus reducing the burden on mental health professionals and the additional treatment costs incurred when patients are diagnosed at a later stage of the disease [9].

In addition, the workflow of Figure 1 could be considered a “good practice” as it supports and facilitates patients in scheduling appointments at the outpatient psychiatric clinic. Through the MMSE and GDS assessments, a concrete pathway without losses in person-hours of the healthcare professionals is created between the PCPs and the Hospital Clinic. Additionally, LPHCU patient waiting time has been reduced since appointments for patients are not scheduled by the patients or their caregivers, but directly from the administrative service of the LPHCU. Moreover, this approach reduces the financial burden for both healthcare institutes and patients, who are seeking the proper treatment, since non-necessary hospital visits do not take place. Additionally, the proposed workflow prevents stigmatization of older adults, taking into account that LPHCU is an environment already familiar to them.

To this end, early recognition, diagnosis, and treatment of mental disorders could play an essential role in the prevention of severe symptoms and overall deterioration. The implementation of a national centralized action plan for the establishment of mental health services in PHC is required that will be conducted in a systematic and continuous way to support all LPHCU. Primary care and mental health policy and practice should be interconnected [22] with the priority to provide efficient training to PCPs and appropriate educational material. The organization of certified programs and adequate training in the field of psychogeriatric care is also of utmost importance.

In this framework, the role of new technologies in PHC could support the prevention and care of mental disorders in older adults. Currently, it was shown that the use of such technologies for the momentary assessment has encouraged older patients to improve their mental health [25]. Specifically, digital frameworks and sensors of vital signals along with mobile applications are employed to not only empower patients to understand better the treatment plan and decisions but also to be closely monitored by their GPs. This enhances the relational continuity, i.e., the ongoing therapeutic relationship between a patient and one or more healthcare physicians or caregivers upon various healthcare events, which results in both the accumulated knowledge of the patient about the health status and the care sensitivity and continuity with respect to the individual needs of the patient [26]. However, several barriers like the lack of prior knowledge applicable to digital technologies require resolving. In this context, a number of research projects funded by the European Union, including SHAPES (Smart & Healthy Ageing through People Engaging in Supportive Systems) [27] and CAPTAIN (Coach Assistant via Projected and Tangible Interface) [28], intend to develop an open ecosystem that will enable the large-scale deployment of digital solutions for healthy and independent living of older adults.

Care for older adults with mental disorders requires sensitivity, as well as observational and relational skills in order to help them achieve and maintain the highest possible level of functioning and wellbeing. Governments, healthcare professionals, and other civil society organizations and stakeholders could all build substantial contributions with the aim to (a) develop efforts for early identification, prevention, and screening; (b) support and empower families, caregivers, and patients; (c) influence the behavior of PCPs and improve the quality of care; (d) include professional counseling for PCPs, as well as continuing professional development and capacity building courses; and (e) establish e-health interventions [29]. Policymakers ought to take decisive action to introduce and scale-up good practices and support mental health in broader social systems, whilst protecting and promoting human rights [9]. All these interventions could be efficiently performed in primary care.

## 5. Limitations and Future Directions

This paper is part of an ongoing study and further data collection. The involvement of a greater sample of older adults is needed to assure the effectiveness of the proposed evidence-informed policymaking in Greece. To this end, more data are expected to be collected throughout the continuation of this study. Furthermore, the COVID-19 crisis has delayed the scheduled training follow-ups along with the participation of other LPHCU in the study. We focus on advancing mental health practices by scaling up the quality of the primary healthcare services.

## Data Availability

Not applicable.
